# A novel ultrathin cholangioscope for endoscopic ultrasound-guided antegrade intervention in patients with Roux-en-Y hepaticojejunostomy

**DOI:** 10.1055/a-2127-4957

**Published:** 2023-08-21

**Authors:** Tadahisa Inoue, Rena Kitano, Mayu Ibusuki, Yuji Kobayashi, Kiyoaki Ito, Masashi Yoneda

**Affiliations:** Department of Gastroenterology, Aichi Medical University, Nagakute, Japan

Cholangitis can occur after biliary reconstructive surgery and is most commonly caused by hepaticojejunostomy anastomotic strictures and bile duct stones 1 . Endoscopic ultrasound-guided antegrade treatment (EUS-AG) has recently been performed in cases where balloon enteroscopy-assisted endoscopic retrograde cholangiopancreatography (BE-ERCP) is difficult 2 . In EUS-AG, cholangioscopy is needed to obtain direct visual information on the anastomosis. However, it is often difficult to insert a cholangioscope in a significantly dilated fistula due to the narrowness of the intrahepatic bile ducts. We report the feasibility of a novel ultrathin cholangioscope (DRES Slim Scope; Japan Lifeline Co., Ltd., Tokyo, Japan) that has almost the same diameter as a standard ERCP catheter ( Fig. 1 ).

**Fig. 1:**
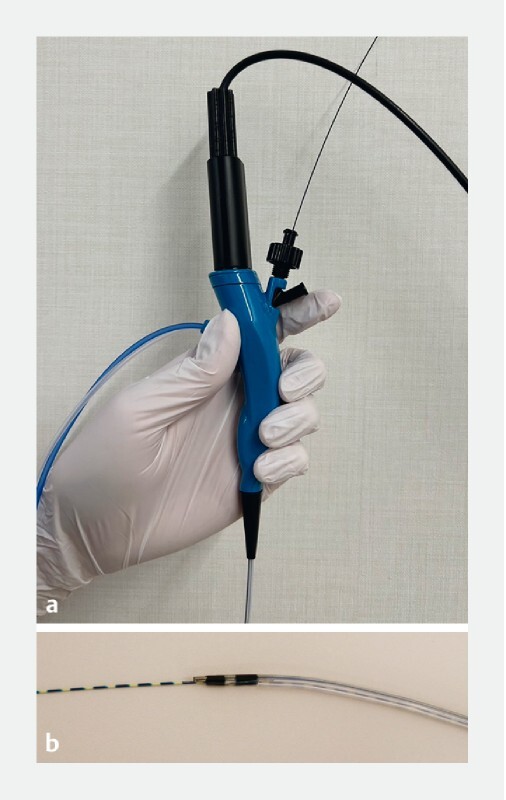
The novel ultrathin cholangioscope ( **a, b** ) is similar in size to a standard endoscopic retrograde cholangiopancreatography catheter. It has a 4.4-Fr tapered tip, a length of 1950 mm, and the compatible guidewire measures 0.025 inches. Independent water supply and suction channels are located 10 mm from the tip, so that simultaneous irrigation and observation can be performed, similarly to conventional large-diameter cholangioscopes.

An 80-year-old man who underwent hepaticojejunostomy with Roux-en-Y reconstruction for cholangiocarcinoma developed cholangitis with intrahepatic bile duct dilation. BE-ERCP was initially performed for biliary drainage and evaluation of the hepaticojejunostomy anastomosis, but the scope failed to reach the anastomosis site. Therefore, we converted to an EUS-guided hepaticogastrostomy and placed a 7-Fr single-pigtail plastic stent from the intrahepatic duct to the stomach. Cholangitis rapidly improved, and no adverse events were noted.

A standard duodenoscope was inserted 2 weeks postoperatively, followed by guidewire insertion from the fistula into the bile duct, alongside the hepaticogastrostomy stent. Subsequently, the stent was removed, and the ultrathin cholangioscope was inserted without any fistula dilation. No stricture was observed at the anastomosis site, and there was no evidence of recurrence. Therefore, balloon cleaning was only performed antegradely without replacement of the stent ( Fig. 2 , Video 1 ). The treatment achieved good clinical course with no adverse outcomes.

**Fig. 2:**
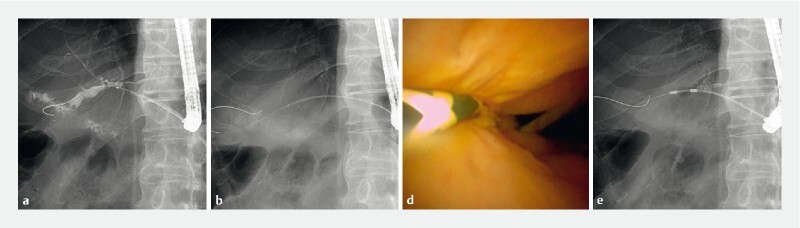
Use of the ultrathin cholangioscope. **a** A 0.025-inch guidewire was inserted into the bile duct, from the fistula, alongside the hepaticogastrostomy stent. **b** Subsequently, the stent was removed, and the ultrathin cholangioscope was inserted without any fistula dilation. **c** No stricture was observed at the anastomosis site, and there was no evidence of recurrence. **d** Therefore, balloon cleaning was only performed antegradely without replacing the stent.

**Video 1** Utility of the novel ultrathin cholangioscope during endoscopic ultrasound-guided antegrade intervention in a patient with Roux-en-Y hepaticojejunostomy.


Although the novel ultrathin cholangioscope does not have a large working channel, its small diameter allows easy insertion and manipulation, and it is less invasive and inexpensive. EUS-AG can be considered a good indication for this novel device.

Endoscopy_UCTN_Code_TTT_1AS_2AD

## Notes

Endoscopy E-Videos

https://eref.thieme.de/e-videos
*E-Videos* is an open access online section of the journal *Endoscopy* , reporting on interesting cases and new techniques in gastroenterological endoscopy. All papers include a high-quality video and are published with a Creative Commons CC-BY license. Endoscopy E-Videos qualify for HINARI discounts and waivers and eligibility is automatically checked during the submission process. We grant 100% waivers to articles whose corresponding authors are based in Group A countries and 50% waivers to those who are based in Group B countries as classified by Research4Life (see: https://www.research4life.org/access/eligibility/ ). This section has its own submission website at https://mc.manuscriptcentral.com/e-videos
